# Escape Game to Promote Students’ Mental Health Outcomes in the Aftermaths of COVID-19 Pandemic: Protocol for a Mixed Methods Study Evaluating a Cocreated Intervention

**DOI:** 10.2196/64068

**Published:** 2025-04-02

**Authors:** David Labrosse, Clara Vié, Mireille Harb, Ilaria Montagni

**Affiliations:** 1 Tricky Bordeaux France; 2 Bordeaux Population Health U1219 Inserm University of Bordeaux Bordeaux France

**Keywords:** Escape game, pilot randomized controlled trial, Covid-19, cocreation, students, mobile phone

## Abstract

**Background:**

The COVID-19 pandemic and the protracted lockdowns have heavily impacted university students’ mental health. Digital Escape Games represent a good means to reach students and propose them solutions for their psychological well-being.

**Objective:**

This study aimed to evaluate a cocreated digital Escape Game on students’ mental health in the aftermath of the COVID-19 pandemic, called EscapeCovid Game. The evaluation of the effectiveness of this stand-alone intervention concerns mental health outcomes (mental health literacy, appraisal and change of beliefs about mental health, management of emotions, and development of coping strategies) and the appreciation and relevance of the game.

**Methods:**

A randomized controlled trial with pre- and posttest data collection (online questionnaires with validated scales) is conducted among 500 students in Bordeaux, France, to evaluate the EscapeCovid Game cocreated with students, researchers, health professionals, and web developers. A subsample of students is randomly selected for responding to a semistructured interview following a mixed methods design. Recruitment is done through mail invitations from student associations and presentations in university classes. Half of the sample of the trial plays the Escape Game, while the other half receives an email with mental health–related information. Within the game, students discuss their personal experiences. The text is further used for the qualitative analyses. The whole study is carried out online.

**Results:**

The EscapeCovid Game has been developed, tested, and finalized by the end of March 2023. As of November 4, 2024, a total of 191 students have answered the baseline questionnaire (90 intervention vs 101 control). A total of 23 students have played the game and 53 are in the control arm. Among participants, by the end of September 20, 2023, twenty were interviewed (10 intervention and 10 control) reaching sample saturation. According to preliminary results, the EscapeCovid Game has had a positive impact on all defined outcomes, while the email has been effective in increasing knowledge on resources available and on coping strategies and meditation techniques. We expect the trial to be completed by the end of June 2025.

**Conclusions:**

The mixed methods findings of this study are due to demonstrate the effectiveness of the EscapeCovid Game in improving students’ mental health outcomes. Preliminary results from the qualitative substudy are promising: in the aftermath of the COVID-19 crisis, this intervention is intended to promote players’ mental health through gamification, knowledge transfer, and a learning-by-doing approach.

**Trial Registration:**

ClinicalTrials.gov NCT06720792; https://clinicaltrials.gov/study/NCT06720792

**International Registered Report Identifier (IRRID):**

DERR1-10.2196/64068

## Introduction

### Background

Mental health problems among young people have skyrocketed with the COVID-19 crisis [1]. Repeated lockdowns and curfews between 2020 and 2021 were the causes of a general mental distress well-documented in the literature [2]. These measures had detrimental and long-lasting psychosocial consequences like acute stress and trauma-related disorders, particularly in specific at-risk populations such as students [3].

University students were in fact concerned as a vulnerable population. Independently from the presence of a pandemic, suicide is the second leading cause of death among them and, by the age of 25 years, 75% of those who will have a mental health disorder have had their first onset [4,5]. During the COVID-19 crisis, students were exposed to supplementary heavy stressors: isolation, classes exclusively online, and uncertainty about their academic and professional future. Several studies have shown how the COVID-19 pandemic and its lockdowns had negatively affected students’ mental health across the world [6,7]. Therefore, it is fundamental to implement mental health promotion and prevention interventions aimed to support students during this crisis. Evaluation of these interventions is a pivotal process in public health research to determine their effectiveness and improve their quality in relation to the targeted population [8].

Among innovative interventions, Escape Games are “live-action team-based games where players discover clues, and solve puzzles [...] in order to accomplish a specific goal, usually escaping from the room, in a limited amount of time” [9]. Escape Games are the digital internet-based online version of Escape rooms. Previous research has proved that Escape Games can increase knowledge on health-related topics using the learning-by-doing technique [10]. Furthermore, gamification is based on a learning by making mistakes approach. Related flow theory states that simple simulation can be perceived as more anxiety-provoking than the purely playful approach which integrates error as a dynamic element.

Compared with other interventions, they are supposed to be more attractive and acceptable for a young audience, especially because of their gamification approach. Gamification relies on immersion through which players feel embedded in the game as several psychosocial determinants (eg, social support, environment) are leveraged. In particular, health-related Escape Games use puzzle solving as a strategy to access health promotion and prevention messages. The step-by-step sequencing and the resolution of the enigmas stimulate the learning loop and over-solicit the cognitive capacity of participants (ie, better awareness of the concerned topic, and better capacity to deconstruct one’s beliefs).

Escape Games can contribute not only to increasing knowledge, but also to behavioral change by providing instruments to act in real-life. For instance, by relying on teamwork, participants can develop the notion of mutual support which could be transposed to their communities. Thus, individual and collective behaviors are at stake. The positive outcomes of Escape Games are boosted by their digitalization: through the internet, Escape Games can reach a larger audience with no time or space limit. This should be even more promising in a period where face-to-face contact is very limited. Success of these interventions can also be fostered if they are cocreated with the target population through a design thinking approach (ie, understanding of users’ experience and needs, brainstorming sessions with several stakeholders, and testing of the tool). Cocreation is a collaborative approach offering various benefits such as enriched insight into problems and increased feasibility and acceptability [11]. We applied this approach to produce the EscapeCovid Game, a stand-alone intervention to promote mental health and prevent psychological diseases among university students in the era of COVID-19.

### The EscapeCovid Game

The EscapeCovid Game is focused on four main outcomes: (1) mental health literacy (knowledge of the concept of mental health, from illnesses to psychological well-being), (2) appraisal and change of beliefs about mental health (destigmatization), (3) management of emotions (their recognition and regulation), and (4) development of coping strategies (building resilience). It addresses specifically anxiety and depression whose symptoms were exacerbated by the COVID-19 crisis including the measures undertaken to limit the spread of the virus (eg, repeated lockdowns and curfews, University closure).

The underpinning theories of the game are the COM-B model of behavior (capability, opportunity, and motivation to change behavior) [12], the Plutchik’s wheel of emotions (visualization of emotions to change behavior) [13], and the Transtheoretical model of behavior change (stages of change, processes of change, levels of change, self-efficacy, and decisional balance [14]).

The EscapeCovid Game takes place in Thomas’ apartment which he shares with another student, Hana. Thomas is a university student and we follow him during a typical day in lockdown. To move from one room to another, players must solve all the puzzles by clicking on the objects spread out in Thomas’ room. At the end of each room scenario, a set of cards is shown, with each card containing a mental health-related message. The cards synthesize the messages transmitted through the puzzles in the room.

The game can be played in groups of 4 players who help each other and discuss using their computer cameras and headphones. All along the game, they are helped by a game guide who explains the rules and answers any questions. The same guide concludes the game session with a final debrief where all participants share their experiences.

The EscapeCovid Game has been cocreated jointly by a team of researchers at the University of Bordeaux and a start-up specialized in the production of both physical and digital Escape Rooms, Tricky, in France. We used the PRODUCES (Problem, Objective, Design, End Users, Cocreators, Evaluation, and Scalability) framework [15] composed of 3 steps. First, we tested the hypothesis that the game was effective in promoting the four mental health-related outcomes. Second, we accumulated evidence on the good functioning of the game. Third, we leveraged the right behavior change mechanisms.

Within this framework, the game was cocreated in particular by 1 project manager from a public health research center, 1 student intern completing a degree in cognitive engineering, 1 game designer, 1 developer specialized in computer science, 1 medical doctor, and 1 psychology researcher. Supplementary data to improve the game was provided by 45 questionnaires on the appreciation and relevance of the game by student players, and qualitative semistructured interviews with 10 students of the same sample. The cocreation of the game is detailed in an article by Labrosse et al [16].

### Objective and Hypotheses

The objective of this study is to evaluate among a population of university students the effectiveness of the EscapeCOVID Game on 4 mental health outcomes: mental health literacy, appraisal and change of beliefs about mental health, management of emotions, and development of coping strategies. The appreciation and relevance of the game are also considered in the overall evaluation of the game. This stand-alone intervention is implemented in the era of the COVID-19 pandemic.

Consequential research hypotheses of the study are (1) compared with a group receiving another classical intervention, after playing the digital Escape Game and having received a debriefing, students remarkably improve their mental health literacy, appraisal, and change of beliefs about mental health, management of emotions, and development of coping strategies; and (2) cocreation enhances appreciation and relevance of the digital Escape Game among university students.

The objective of this study is original since, to the best of our knowledge, no Escape Game on students’ mental health outcomes has been developed before following a cocreation approach and evaluated through mixed-methods to prove its effectiveness.

## Methods

### Evaluation of the EscapeCovid Game

#### Mixed Methods Research Design

The EscapeCovid Game is evaluated through a mixed-methods approach using exclusively online data from a Randomized Controlled Trial (RCT) including 2 web-based questionnaires, and video interviewing. Within the mixed methods framework, we apply the embedded design [17]. According to this design, the quantitative and the qualitative data are collected simultaneously, but the qualitative data is embedded within the quantitative data. This means that the data from the questionnaires is prominent, but we still need to understand how the qualitative data further explains the effectiveness of the intervention.

As for the cocreation of the Escape Game, all tasks of the evaluation are conducted jointly by the university research team and the private start-up, thus proving the sharing of skills and the management of potential risks and their solutions in a combined effort. In particular, the research team is in charge of the collection and analyses of the data, while the start-up handles logistics including the organization of the game sessions. The postulate of the start-up is that a product can be really evaluated as significantly efficient in the serious game industry mostly or exclusively through the collaboration with a public research laboratory.

#### The Randomized Controlled Trial

We implement a rigorous RCT design using pre- and postintervention data coming from online self-administered questionnaires. The sample must be of at least 500 students at the University of Bordeaux. We estimated a sample size of 500 participants for the 2 arms: statistical power=.80, Cronbach α=.05, Cohen *d*=0.5, and 40% attrition with a 2:1 allocation ratio for the intervention group.

At study entry, the 500 students complete the online baseline questionnaire. Among them, 250 students play the EscapeCovid Game while 250 students receive an email with tips and resources for managing their stress during the COVID-19 pandemic. Participants are randomized so that the 2 groups are similar in terms of sex, age, and field of study (stratified balanced randomization). Participants are informed from the beginning of the study that they can be allocated to either the intervention of interest or the comparator. We tell students in the control arm that they are in a waiting list and that they can play the game once the experiment is concluded.

Inclusion is stopped when at least 500 participants have completed at least 3 quarters of each questionnaire, and 250 have played the game until the end. The statistician and the project manager of the EscapeCovid study are in charge of generating the random allocation sequence, enrolling participants, and assign them to one of the 2 arms.

Briefly, the 250 students of the intervention arm (1) complete the online baseline questionnaire, (2) play the EscapeCovid Game at their home within the week after the online baseline questionnaire—a link to the game is given at the end of the questionnaire, and (3) complete the same online questionnaire of the baseline plus some items on the appreciation of the game (satisfaction survey) 2 days after having played the game. After some tests with Tricky staff, the game duration is estimated at about 2 hours, from the online registration with the creation of an account, until the end of the debrief. Instructions to connect and play online are provided in an email sent by the start-up to the student of the intervention arm 2 days before the game session.

The 250 students of the control arm will (1) complete the baseline online questionnaire, (2) receive the same day an email with tips and resources to manage their stress during the COVID-19 pandemic, and (3) complete the same internet-based questionnaire of the baseline 2 days after having received the email with tips and resources.

Students can complete all steps of the evaluation from anywhere using a smartphone or a computer. However, they can play the game exclusively from home, considering the need for a stable internet connection and a performance computer.

#### Internet-Based Interviews

There are two sources of qualitative data: (1) semistructured interviews after the intervention with students from both arms and (2) open discussion with the game guide at the end of the game session. Concerning the first source of qualitative data, 20 students were randomly selected among the 2 groups (10 for the intervention arm, and 10 for the control arm) to respond to a semistructured interview. For the discussion embedded in the game, the text produced during the debrief is recorded and analyzed so that the developers of the start-up can work on an algorithm for building a chatbot messenger capable of providing tailored advice to gamers, replacing the game guide. Machine learning will be used for this purpose. In terms of simplicity and feasibility, the sample is recruited on the sole criterion of sex, 50% of girls, through sample saturation. The interviews have been conducted remotely by a public health researcher through an internet-based interview tool. Students are recommended to make the interview at home.

#### Recruitment of Study Population

Inclusion criteria are (1) being a student at the University of Bordeaux, (2) being aged ≥18 years, (3) understanding, speaking, and writing French, and (4) having given the electronic consent to be enrolled in the study. We consider that students have a sufficient level of computer literacy for playing the game as only few basic skills are required: knowing how to navigate the web, keyboarding, and typing. These skills are essential for any student from any field of study and represent a prerequisite for the undergraduate and graduate programs at the University of Bordeaux.

Students are invited through the social media of the study partners (University of Bordeaux and Tricky), posts in the Facebook groups of more than 100 student associations, and mailing lists from the University of Bordeaux. The associations are manually selected from a list provided by the University of Bordeaux to cover the different fields of study. Overall, we plan to reach about 10,000 students which is a substantial number considering potential very low enrollment and retention rates. The invitations by email are sent to a maximum of 500 recipients at a time, so that we can proceed step by step with the inclusion of participants and the organization and schedule of the game sessions. The invitations will be sent until we reach a sample of a minimum number of 500 students.

The recruitment is exclusively web-based. Clicking on the link displayed on social media posts or on the text of the email, students will access an online form to enter the study.

The inclusion of participants to the RCT will be conducted based on a continuous monitoring through statistical analysis. Data on sex are available only for males and females, since information on gender is not reported in the University records. The year of study goes from 1 (first year of university) to >8 (corresponding to a doctoral degree or a diploma in medicine). The fields of study offered by the University of Bordeaux are more than 50,000 and are categorized in Health Studies, Law and Economics, Technical Sciences, and Human and Social Sciences.

Finally, a maximum of 5 reminders will be sent through email to all 500 participants for the completion of the second questionnaire. For the intervention group, an additional maximum of 5 reminders will be sent to schedule a game session and eventually play the game. An additional maximum of 5 reminders will be sent to the students randomly selected to be interviewed. If after the 5 reminders per phase of the study, the student does not reply, we will proceed with the invitation of new students from the contacts we have from the University and attached associations.

The participation to the study is completely voluntary and all students are rewarded with a €20 (US $21.59) gift card.

#### Data Collection Tools

The pre and postintervention questionnaires are administered through the Sphinx software (Georg Brandl) distributing surveys on all media (web, smartphone, QR code, and SMS). The data are automatically imported in a Microsoft Excel file.

The first questionnaire is composed of 112 items. It includes validated scales measuring mental health literacy (French Mental Health Literacy Scale [MHLS-FR]) [18], beliefs about mental health (Health Belief Model [HBM]) [19], emotions (Plutchik’s wheel of emotions) [20], Coping Strategies (Ways of coping questionnaire) [21], Depression (Patient Health Questionnaire-9 [PHQ-9]) [22], Anxiety (Generalized Anxiety Disorder-7 Scale [GAD-7]) [23], Stress (Perceived Stress Scale [PSS4]) [24], and Help-seeking actions (Kessler Psychological Distress Scale in help-seeking youth [K6]) [25]. Sociodemographic information (email address, gender, date of birth, field of study, year of study, and self-perceived health) are also collected to assess the representativeness of the sample (stratifications by sex, age, field of study etc). A question specifically asks the gender giving students the possibility to indicate whether they belong to the LGBT (lesbian, gay, bisexual, and transgender) category (considering the confidentiality of this data). The question of gender as a determinant of mental health will be important to analyze the results considering this confounding variable if the number of individuals is sufficient. The questionnaire is developed by several researchers through different stages of drafts and reviews, following a structured survey construction method in 5 steps [26]. Operational staff members from the team of the coordinator and a group of students pretest the questionnaire for readability, comprehensibility, and face validity. The response modalities are varied: multiple choice, binary responses, Likert scales, and visual analog scales. Mental health indicators and appreciation and relevance results are the main outcomes of the study. All items provided the nonresponse option “not applicable,” “I don’t know,” and “I do not want to answer.” Respondents are required to select an option from a predefined list. The questionnaire is not validated if at least one question is not answered. Respondents are not able to review and change their answers, but they can ask the principal investigator for a document with exclusively their answers. Corrections cannot be made manually in the database in order to avoid any further error.

The second questionnaire is the same, but it does not include the sociodemographic items. It contains new items on the appreciation and relevance of either the game or the email (satisfaction survey). These items are created ad hoc, but are also inspired by existing instruments evaluating digital health interventions (eg, items by Fadda et al [27]). The questionnaires, composed of both validated scales, sociodemographic items, and satisfaction survey questions, have been developed by 1 researcher and 1 project manager in public health. A pool of researchers in psychology and in statistics have reviewed the questionnaire to assess its readability, understanding, and relevance. The questionnaires have been tested on the internet by 10 students of the School of Public Health of the University of Bordeaux. Testers reported some technical errors, that has bugs and repetitions of items, and suggested different wording for making the questionnaires more adapted to a young target population between 18 and 25 years of age. The questionnaires were constructed following the CHERRIES (Checklist for Reporting Results of Internet E-Surveys) [28]. For both questionnaires, outcomes are self-assessed. The questionnaires are available in Multimedia Appendix 1.

Qualitative data from the semistructured interviews are collected through an ad hoc grid including questions on the impact of the game or email on mental health–related knowledge, on the access to mental health care services and the attitudes toward mental illnesses or stigmatization. Participants are particularly asked to evaluate whether the game is adapted to their needs and to discuss the cocreation approach as a means to produce tailored interventions. The main objective of these interviews is to assess the appreciation and the relevance of the game. The interview grids are available in Multimedia Appendix 2.

Questions addressed to the control group allow to capture the utility of the contents of the email (eg, list of mental health care services and a short description of mental health problems) and to compare the contribution of each tool to knowledge about mental health. Qualitative data form the debrief correspond to the free discussion between the players and the game guide. The latter follows a script and makes questions on the specific topics covered by the game: overall recognition and management of the emotions; symptoms of depression, stress and anxiety; relevance of mental health in daily life; and sources available in the case of a mental health problem.

The semistructured interviews were conducted by a public health researcher trained in qualitative data analyses. The project manager analyzed the same dataset using the triangulation approach.

The quality of both quantitative and qualitative data is ensured by keeping documentation accurate, that are clean databases and well-stored texts. Ensuring ethical standards is a further guarantee of the quality of this study.

### Data Analyses

#### Overall Mixed Methods Analyses

First, we will conduct statistical analyses on quantitative data (descriptive statistics, modeling, and exploratory data analysis) processed from the RCT; second, we have analyzed the discourse of qualitative data (thematic analysis based on a predefined interview guide); and third, we will couple the analysis of both types of data. In particular, we will integrate quantitative and qualitative data for the purposes of convergence, contextualization, and expansion to gain a detailed understanding of the evaluation and impact of the game.

#### Quantitative Data Analyses

For the data from the RCT, we will compare the answers between exposed and nonexposed students (intergroup) and measure the change in their answers within the intervention group before and after playing the game and debriefing (intragroup). Our primary analysis will be based on intention-to-treat at the individual level using linear regression comparing the outcomes of the intervention and the control arm. We will measure our primary outcomes in terms of absolute scores and as a percentage of the total possible score. We will control baseline covariates: (1) gender, (2) age, (3) field of study, (4) year of study, and (5) self-perceived health. We will analyze the data from all participants only if at least 3 quarters of the questions are completed. We consider that some users will not go through all questionnaire pages. Missing values will be imputed by replacing them with the mean or median values of the dataset at large, or some similar summary statistics.

#### Qualitative Data Analyses

Concerning qualitative data from the semistructured interviews (intervention and control groups), they have been analyzed through content analysis: we have identified patterns across collected texts which are coded according to key elements. These can include, for instance, reported advantages and drawbacks of the game, or players’ opinions on the usefulness of the game in general. The semistructured interviews are either video or audio recorded depending on the availability of the participants, during the lunch break or in the late evening after the classes. In-depth analyses were conducted using a reflexive approach so as to appraise and evaluate how subjectivity and context influence the research processes, especially during the interpretation of the data on intimate topics like mental health. Before the association with the quantitative data, the texts will be cross-analyzed to identify similarities and differences between the experience with either the escape game or the email.

Data from the debrief at the end of the game were recorded automatically within the game. The texts were collated in a single document which was used to generate repeated and automated messages. Through artificial intelligence and, more specifically, the machine-assisted topic analysis (MATA), the human conversation will be processed to simulate answers to players’ requests. The outcome will correspond to a chatbot, which will a computer program that will be able to animate a discussion and provide tips concerning mental health–related issues.

### Ethical Considerations

The study has received the French identifying number (ID-RCB) 2024-A00436-41 from the National Agency for the Safety of Medicines and Health Products. This number is compulsory for registering projects on human subjects in France.

The Ethics and Health Research Center of the University of Bordeaux approved the study (2024-A00436-41). The procedures are in accordance with the Helsinki Declaration of 1975, as revised in 2024.

It is compulsory that students carefully read the consent form and sign it electronically (by clicking on the “I agree button”) in order to participate to all steps of the study and share their data collected through questionnaires, interviews, and during the game. The consent form includes all the details of the study: (1) principal investigator, (2) rationale, (3) objectives, (4) methods, (5) length, (6) conditions of the storage data and analyses, and (7) expected impact. Students access directly to the consent form when they click on the link in the social media posts and the study invitation email. Participants are given the chance to opt out.

All confidential information, including participants’ contact details or other sensitive data like self-reported mental health status will only be accessible to authorized staff members from the study. Thus, information is quasi-anonymous for the proper running of the study. In fact, the email address is necessary to organize the game sessions, to send the links to the web-based questionnaires and to schedule the internet-based interview. The phone number is also required to resolicit the participants, only once, for the organization of the game session. We guarantee the noncommercial use of the email addresses and the phone numbers.

Overall, participants are informed that they are not blinded.

All data, including the sensitive ones, are imported automatically from the software Sphinx to Excel. The document is protected by a password known only to the principal investigator and the statistician of the research team. The data will be destroyed 2 years after the last article based on this study is published. The collection, storage, and analysis of the data comply with the European General Data Protection Regulation (GDPR).

As already mentioned, students will receive US $21.5 gift cards for their participation to the study. The authors have filled the CONSORT-EHEALTH (Consolidated Standards of Reporting Trials of Electronic and Mobile Health Applications and Online Telehealth) form V1.6.1 (Multimedia Appendix 3).

## Results

### Key Dates of the Study

**Figure 1:**
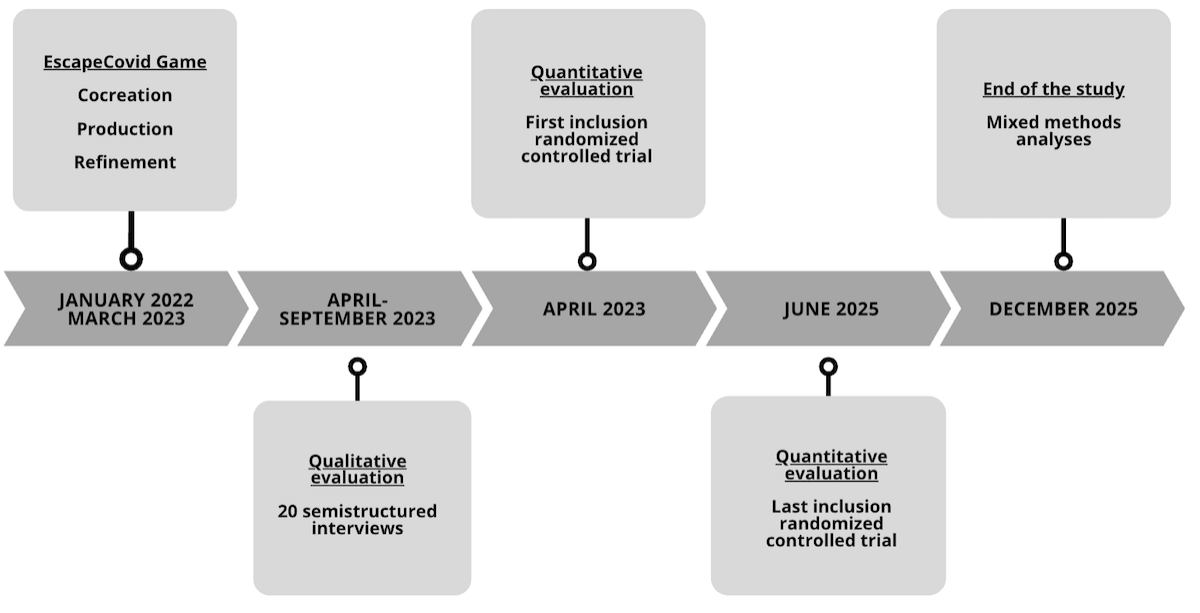
Timeline of the phases of the study.

The study received funding by the French National Research Agency in January 2022. The production of the EscapeCovid Game ended in March 2023. The cocreation process, the test, and the refinement of the intervention lasted 15 months. For the evaluation of the finalized game, the collection and analyses of qualitative data through semistructured interviews has been completed by September 2023. The end of the evaluation phase through the RCT is scheduled to be completed by the end of June 2025. The results of the mixed methods analyses are expected to be published by the end of December 2025. The timeline of the study is illustrated in Figure 1.

### Study Population

The recruitment started in December 2023.

As of November 4, 2024, a total of 191 students have answered the baseline questionnaire and have been randomly assigned to one of the 2 arms, 90 intervention versus 101 control. A total of 23 students have played the EscapeCovid Game (intervention arm) and 53 are in the control arm. Specifically, 54% (103/191) of the participants at the baseline are students in health care disciplines; 11.5% (22/191) in sciences and technologies; and the remaining participants study other subjects 34.5% (66/191) including psychology, law, and so on.

Concerning qualitative data, 20 students (10 for each arm) have been interviewed on the appreciation, relevance, and acceptability of either the game or the email. Students of the game arm were between 18 and 28 years of age. There were 9 females and 1 male. Their year of study ranged from the first year of their bachelor’s degree to the first year of their doctoral studies. Fields of study included medicine, chemistry, physics, biology, sociology, speech therapy, and political science. Students of the control arm were between 18 years and 28 years old. Their year of study ranged from the second year of their Bachelor’s to the third year of their doctoral studies. Fields of study included medicine, physics, nursing and marketing.

### Preliminary Quantitative and Qualitative Results

As of November 4, 2024, the sample size of the trial is too small for providing robust data and for reaching significance level.

As far as qualitative data are concerned, we completed all 20 interviews. According to the students from the intervention harm, the game was appealing and fun. The use of gamification to communicate mental health-related messages was appreciated.

I like the initiative of doing something more fun, more educational than just distributing information, which is also a way in itself, we agree, but I like the way of trying to learn.

Student 7–Male

The game was described as “innovative,” “unique,” “instructive,” and “beneficial” considering its alternative approach to conventional public health interventions, for example, educational materials, and social marketing campaigns.

The notion of stigmatization emerged. Attached outcome was the appraisal and change of beliefs about mental health.

I found it very interesting that we raise awareness about mental health because I have the impression that it is something that we talk about more than before but there is still a taboo behind it.

Student 2–Female

The game also contributed to increase participants’ mental health literacy.

It gave me knowledge I didnot have about mental health.

Student 1–Male

The impact of the intervention on support recognition and management of emotions was also positive.

The way in which the symptoms or rather really what the person thinks to themselves are well represented and so it really allows us to understand what is going on in the person's head and also to know the symptoms and emotions. So it's very interesting, rather than just listening to someone talk about it and having a slide, it's much better.

Student 9-Female

The other study outcome (teach positive coping strategies) was also reported:

Well, you see that I have to do things that finally make us happy and otherwise, not to work too late, to take a break to eat, to see my friends and that's it and from time to time go out when we need to get some fresh air.

Student 5-Male

However, the students also declared that the content of the game should be enriched with the presentation of more coping strategies in order to support the player’s psychological well-being. Thus, the impact of the intervention should be directly on the mental health of the players and not only on their knowledge and beliefs.

Not particularly, I don’t think so, it was more focused on knowing emotions rather than how to manage them.

Student 1-Male

In comparison to the game, the informative email was not an immersive experience and was not expected to influence students’ cognition, affect, potential, and health behavior. However, it provided useful tips to students from the control arm, including the following: a list of existing sources in case of mental health distress (eg, helplines and free-of-charge consultations with a psychology based at the University of Bordeaux); breathing techniques and meditation; and mental health problems explained with accessible but accurate terminology.

It gave me knowledge I didn't have, about the numbers to contact in case of need [...] we can't know them all by heart and I think that having them all written down in an email or in a document, well I think that it can help us because not everyone knows where to refer and where to go in case of need.

Student 1-Male

The usefulness of the email consisted in the fact that it could be used as a memorandum and repertoire of the basics of mental health literacy: knowing that mental health includes both positive mental health and the absence of a mental health problem; knowing the signs of the most common mental health problems (depression, anxiety, and stress); recognizing and managing emotions; learning coping strategies; and having a list of the most known services in case of a mental health distress.

As of November 4, 2024, data from the game debriefs is not transcribed and cannot be analyzed.

## Discussion

### Principal Findings

This paper outlines the protocol for the evaluation of a cocreated Escape Game on students’ mental health outcomes contributing to prevent and manage mental health diseases and promoting psychological well-being: mental health literacy, appraisal and change of beliefs about mental health, management of emotions, and development of coping strategies. The evaluation is conducted through mixed methods, combining the quantitative results of a RCT with the qualitative results of semistructured interviews.

Concerning quantitative, as of November 4, 2024, the recruited sample is too small to make inferences on the impact of the game. Actually, recruiting students is the hardest challenge of the trial. All along the study, we have observed that the most successful recruitment strategies are: offering gift cards to students completing the whole study (baseline questionnaire, intervention or control arm, and repeated questionnaire); counting on the support of student associations promoting the study among members; and involving professors who present the study during their classes. We will reinforce these strategies to attract and retain students in the trial.

As for the qualitative data, the recruitment of students was far easier and we rapidly interviewed 20 participants by September 2023. The comparison of the responses across the 2 groups, game versus email, showed that gamification improved the retention of messages, especially in terms of recognition and management of emotions. On the other hand, email was considered useful as a memorandum of mental health–related information. In particular, students appreciated the list of available services that they could keep and save for contacting professionals in case of need.

Based on preliminary results, these 2 distinct tools, the EscapeCovid Game, and the informative email, could prove to be mutually complementary in promoting student mental health. By consolidating the specific advantages of each approach while mitigating their respective limitations, we can aspire to a more holistic and informed approach to strengthening psychological well-being within the student community.

### Comparison to Previous Work

Recently, interventional research took an interest in digital games to promote positive well-being and prevent mental health problems. This might be due to the emerging mental health challenges because of the COVID-19 pandemic [29]. A scoping review of 16 articles has identified positive effects of gamified interventions, with beneficial consequences for psychological well-being and depressive symptoms [30]. Three articles focused specifically on university students, but described interventions were gamified mobile or web applications, and did not include Escape Games.

Overall, Escape Games addressed to university students are mostly used as educational tools aimed at guiding learners toward achieving specific learning outcomes through a game-based design [31]. Concerning mental health, the literature reports 1 game addressed to students to improve their knowledge on psychological well-being and mental illnesses, but it is an Escape Room exclusively on site [32].

It is essential to support this population and reinforce their psychological status and resilience since they will be the future workforce. They will especially be in charge of rebuilding the economy of the country after this huge crisis.

### Strengths and Limitations

The robust evaluation process of this study will provide evidence of the effectiveness of one of the few digital gamified interventions addressed to students’ mental health. This will be among the first promising Escape Games produced jointly by a public university research team and a private start-up. The collaboration between these 2 institutions is an added value guaranteeing that, on the one hand, the intervention is well-designed and functioning, and that, on the other hand, researchers have accurately assessed its performance.

The main strength of the evaluation process in that relies on mixed methods. This approach can balance out the limitations of each method (questionnaires from an RCT and qualitative data from semistructured interviews and game briefing); it can lead to stronger evidence and reliability; and it can provide more details than each individual method. Concerning quantitative data, mixed methods limit straightforward and stereotyped interpretations, and reduce ambiguity and misunderstanding.

On the other hand, the limitations of qualitative data are mitigated by a rigorous quantitative approach: coding of the text in structured interview grids, word-by-word data management, and detailed records of data collection and analysis like in an audit trial [33].

Thus, mixed methods can increase the trustworthiness, transferability, dependability, and confirmability of the thick description of combined quantitative and qualitative data.

This study is primarily limited by the low participation of students and inclusion at the baseline. This might be due to the fact that the game lasts more than 1 hour and requires a good computer operating system, as it is already stated in the interviews. Students’ agenda are really busy and their workload is heavy. As suggested, new structured communication strategies would improve the participant recruitment. Furthermore, the inclusion in the study is completely voluntary and, considering that we do not use quota sampling because of feasibility, results may not be generalizable to students in Bordeaux and to French students in particular.

Another barrier is the correct implementation of a complex evaluation through an RCT and mixed methods design with the collection of several data. Researchers involved in the study have renowned experience on this type of methodology and are keen to ensure the smoothness of the evaluation process. As far as technical barriers are concerned, the development of the digital Escape Game has to overcome potential IT challenges. The final product will need to be both high-tech and appealing, mixing solid technology with a modern design and avoiding bugs. IT developers of the start-up of the study have already proven their competencies and skills for that.

### Future Directions

If the results of the study show the impact of the intervention on mental health outcomes (mental health literacy, appraisal and change of beliefs about mental health, management of emotions, and development of coping strategies) among students, the game will be commercialized and sold to universities for its deployment on the campuses across France. The evaluation process is meant to guarantee that the game can be diffused with an economically sustainable model defined by the start-up. In particular, this study will help the start-up to define a “gold standard” for selling other similar interventions.

The promising results of the study open the way to crucial questions: how can we secure funding and optimize the economic efficiency of the EscapeCovid Game in order to guarantee its sustainability and make it a mental health promotion tool, accessible to a wide range of students from diverse backgrounds? For instance, using artificial intelligence, it will be possible to make the intervention autonomous with automatized debriefing. This will make the game more sustainable cutting the costs of the salary of the game guide.

Furthermore, who will be the legal owner of the EscapeCovid Game? Intellectual property must be deeply discussed to ensure the shared collaboration of the partners to create the game before its real commercialization. In order to overcome any potential disagreement, the roles of all parties must be clearly stated in a business partnership agreement.

Thus, the protocol of this study describes how to evaluate with a robust methodology a game on students’ mental health as an example for producing further effective interventions on the same topic. Based on evidence, these interventions can be commercialized through an economically sustainable model.

Finally, this protocol presents a study whose objectives are both research (studying several indicators of young people’s mental health during the COVID-19 pandemic)- and business-oriented (producing an evidence-based tool for further deployment).

## Appendix

Multimedia Appendix 1 Original Questionnaires for the quantitative evaluation of the EscapeCovid game.

Multimedia Appendix 2 Original interview guides for the qualitative evaluation of the EscapeCovid game.

Multimedia Appendix 3 CONSORT-EHEALTH (Consolidated Standards of Reporting Trials of Electronic and Mobile Health Applications and Online Telehealth) checklist.

## Abbreviations

CHERRIES: Checklist for Reporting Results of Internet E-Surveys
COM-B: capability, opportunity, and motivation to change behavior
CONSORT-EHEALTH: Consolidated Standards of Reporting Trials of Electronic and Mobile Health Applications and Online Telehealth
GAD-7: Generalized Anxiety Disorder-7 Scale
GDPR: General Data Protection Regulation
HBM: Health Belief Model
K6: Kessler Psychological Distress Scale
LGBT: lesbian, gay, bisexual, and transgender
MATA: machine-assisted topic analysis
MHLS-FR: French Mental Health Literacy Scale
PHQ-9: Patient Health Questionnaire-9
PRODUCES: Problem, Objective, Design, End Users, Cocreators, Evaluation, and Scalability
PSS4: Perceived Stress Scale
RCT: randomized controlled trial

## Data Availability

The text of fully transcribed interviews is available from the corresponding author upon request. Quantitative data from the first participants to the randomized controlled trial are preliminary and cannot be shared until the study is concluded.
