# Supplementary material for: Escape Game to Promote Students’ Mental Health Outcomes in the Aftermaths of COVID-19 Pandemic: Protocol for a Mixed Methods Study Evaluating a Cocreated Intervention
Source: JMIR Res Protoc. 2025 Apr 2;14:e64068. doi: 10.2196/64068 (PMC12004027; doi:10.2196/64068)
Supplement: Multimedia Appendix 1 [file resprot_v14i1e64068_app1.pdf]

Appendix 1

ESCAPECOVID – Questionnaire

Nr. questionnaire CODE_QUEST

1. BLOC 1 : Qui êtes-vous ?

1.1 Adresse email

1.2 Genre : F/M/Autre GENRE

1.3 Mois et année de naissance : ../…. MOIS_AN_NAISS

1.4 Filière d'études : FILIERE

Psychologie 1
Autres études en Sciences Humaines et Sociales, Lettres, Langues, Art 2
Sciences, Technique, Ingénierie, Informatique 3
Droit, Économie, Gestion, Finance, Sciences Politiques, Comptabilité 4
Médecine 5
Autres études en Santé 6

1.5 Année d'études : ANNEE

1ère 1
2ème 2
3ème 3
Plus que 3ème 4

1.6 Comment vous sentez-vous en ce moment ? SENTIR

Très bien 1
Bien 2
Moyen 3
Mal 4
Très mal 4

Au cours des 14 derniers jours, à quelle fréquence avez-vous été dérangé.e par les problèmes suivants ? PHQ9

	Presque jamais 1	Plusieurs jours 2	Plus de la moitié du temps 3	Presque tous les jours 4
**1.7 Peu d'intérêt ou de plaisir à faire les choses** PHQ9-1				
**1.8 Se sentir triste, déprimé.e ou désespéré.e** PHQ9-2				
**1.9 Difficultés à d'endormir ou à rester endormi.e, ou trop dormir** PHQ9-3				
**1.10 Se sentir fatigué.e ou avoir peu d'énergie** PHQ9-4				

**1.11 Peu d'appétit ou trop manger** PHQ9-5				
**1.12 Mauvaise perception de vous- même - ou vous pensez que vous êtes un perdant ou que vous n'avez pas satisfait vos propres attentes ou celles de votre famille** PHQ9-6				
**1.13 Difficultés à se concentrer sur des choses telles que lire le journal ou regarder la télévision** PHQ9-7				
**1.14 Vous bougez ou parlez si lentement que les autres personnes ont pu le remarquer. Ou au contraire – vous êtes si agité.e que vous bougez beaucoup plus que d'habitude** PHQ9-8				
**1.15 Vous avez pensé que vous seriez mieux mort(e) ou vous avez pensé à vous blesser d'une façon ou d'une autre** PHQ9-9				

Au cours des 14 derniers jours, à quelle fréquence avez-vous été gêné.e par les problèmes suivants ? GAD7

	Presque jamais 1	Plusieurs jours 2	Plus de la moitié du temps 3	Presque tous les jours 4
**1.16 Un sentiment de nervosité, d'anxiété ou de tension** GAD7				
**1.17 Une incapacité à arrêter de s'inquiéter ou à contrôler ses inquiétudes** GAD7-2				
**1.18 Une inquiétude excessive à propos de différentes choses** GAD7-3				
**1.19 Des difficultés à me détendre** GAD7-4				
**1.20 Une agitation telle qu'il m'est difficile de tenir en place** GAD7-5				
**1.21 Une tendance à être facilement contrarié.e ou irritable** GAD7-6				
**1.22 Un sentiment de peur comme si quelque chose de terrible risquait de se produire** GAD7-7				

Au cours du dernier mois, diriez-vous que : PSS4

	Jamais 1	Rarement 2	Quelquefois 3	Souvent 4	Très souvent 5

**1.23 Il vous a semblé difficile de maîtriser les choses importantes de la vie** PSS4-1					
**1.24 Vous vous êtes senti.e confiant.e dans vos capacités à prendre en mains vos problèmes personnels** PSS4-2					
**1.25 Vous avez senti que les choses allaient comme vous vouliez** PSS4-3					
**1.26 Vous avez trouvé que les difficultés s'accumulaient à tel point que vous ne pouviez pas les contrôler** PSS4-4					

2. MENTAL HEALTH LITERACY (Mental Health Literacy Scale - France)

2.1. Si quelqu'un présente des inquiétudes excessives et difficiles à contrôler, accompagnées de symptômes physiques comme de la tension musculaire et de la fatigue, diriez-vous qu'il est atteint d'anxiété généralisée ? ANX_GEN

Très improbable 1 *Improbable* 2 *Probable* 3 *Très probable* 4

2.2. Diriez-vous que l'agoraphobie se caractérise par une anxiété concernant différentes situations (p. ex. un endroit où il y a beaucoup de personnes) dans lesquelles il pourrait être difficile ou gênant de s'échapper ? AGORA

Très improbable 1 *Improbable* 2 *Probable* 3 *Très probable* 4

2.3. Diriez-vous que quelqu'un ayant une accoutumance physique et psychologique aux drogues (et devant prendre des doses plus fortes pour ressentir le même effet) peut être diagnostiqué comme étant dépendant aux drogues ? DRUG

Très improbable 1 *Improbable* 2 *Probable* 3 *Très probable* 4

2.4. Diriez-vous qu'en général, en France, les femmes sont PLUS à risque que les hommes de souffrir de maladies mentales, quelle qu'elles soient ? FEM_RIS_MAL

Très improbable 1 *Improbable* 2 *Probable* 3 *Très probable* 4

2.5. Diriez-vous qu'en général, en France, les hommes sont PLUS à risque que les femmes de souffrir de troubles anxieux, quel qu'ils soient ? HOM_RIS_ANX

Très improbable 1 *Improbable* 2 *Probable* 3 *Très probable* 4

2.6. Les professionnels de la santé mentale sont tenus à la confidentialité et ils ne peuvent pas la rompre quel que soit le niveau de danger. CONFI_DANG_IMM

Très improbable 1 *Improbable* 2 *Probable* 3 *Très probable* 4

Veuillez indiquer dans quelle mesure vous êtes d'accord avec les affirmations suivantes, le terme « maladies mentales » voulant dire qu'elles nécessitent la prise d'un traitement ou le suivi d'une thérapie (dépression, bipolarité, troubles de la personnalité, etc.) :

	Pas du tout d'accord 1	Pas d'accord 2	Pas d'avis 3	D'accord 4	Tout à fait d'accord 5
**2.7 Je sais où trouver des informations sur les maladies mentales** INFO_MAL					
**2.8 Je suis à l'aise avec l'utilisation d'un ordinateur ou d'un téléphone pour trouver des informations sur la santé mentale** ORDI_INFO					
**2.9 Je suis à l'aide pour poser des questions sur les maladies mentales à des professionnels de santé (p.ex. médecin généraliste)** QUES_MAL					
**2.10 Je sais que je peux accéder à des ressources (p.ex. professionnels de santé, Internet, amis) que je peux mobiliser pour obtenir des informations sur les maladies mentales** RESS_INFO					

Veuillez indiquer dans quelle mesure vous êtes d'accord avec les affirmations suivantes sachant qu'il est question de maladies mentales dans leur globalité, nécessitant la prise d'un traitement ou le suivi d'une thérapie (trouble de l'anxiété généralisée, dépression, bipolarité, troubles de la personnalité, etc.) :

	Pas du tout d'accord 1	Pas d'accord 2	Pas d'avis 3	D'accord 4	Tout à fait d'accord 5
**2.11 Il est préférable de ne pas fréquenter les personnes atteintes d'une maladie mentale afin de ne pas en développer une** FREQ_DEV					
**2.12 Consulter un professionnel de la santé mentale signifie que vous n'êtes pas assez fort pour gérer vous-mêmes vos difficultés** PAS_FORT					
**2.13 Consulter un professionnel de la santé mentale signifie que vous n'êtes pas assez fort pour gérer vous-mêmes vos difficultés** NO_AID					
**2.14 Je crois que le traitement d'une maladie mentale, prescrit par un professionnel de la santé mentale, ne serait pas efficace** TRAIT_PAS_EFF					

Veuillez indiquer dans quelle mesure vous êtes d'accord avec les affirmations suivantes sachant qu'il est question de maladies mentales dans leur globalité, nécessitant la prise d'un traitement ou le suivi d'une thérapie (trouble de l'anxiété généralisée, dépression, bipolarité, troubles de la personnalité, etc.) :

	Pas du tout prêt 1	Probablement pas prêt 2	Pas d'avis 3	Probablement prêt 4	Tout à fait prêt 5
**2.15 Dans quelle mesure seriez-vous prêt.e à habiter à côté d'une personne atteinte d'une maladie mentale ?** HAB_MAL					
**2.16 Dans quelle mesure seriez-vous prêt.e à passer une soirée à discuter avec une personne atteinte d'une maladie mentale ?** DISC_MAL					
**2.17 Dans quelle mesure seriez-vous prêt.e à devenir ami avec une personne atteinte d'une maladie mentale ?** AMI_MAL					
**2.18 Dans quelle mesure seriez-vous prêt.e à travailler en étroite collaboration avec une personne atteinte d'une maladie**					

mentale ? TRAV_MAL					
2.19 Dans quelle mesure seriez-vous prêt.e à ce qu'un membre de votre famille se marie avec une personne atteinte d'une maladie mentale ? MARI_MAL					
2.20 Dans quelle mesure seriez-vous prêt.e à voter pour un politicien si vous saviez qu'il a souffert d'une maladie mentale ? POLI_MAL					
2.21 Si vous étiez chef d'entreprise, dans quelle mesure seriez-vous prêt.e à embaucher quelqu'un si vous savez qu'il souffre d'une maladie mentale ? EMB_MAL					

3 HEALTH BELIEF MODEL

Susceptibilité perçue

3.1 Je suis préoccupé.e par mon risque d'avoir un problème de santé mentale SUS1
Très en accord 1 */ En accord* 2 */ Ni d'accord ni pas d'accord* 3 */ Pas d'accord* 4 */ Pas du tout d'accord* 5

3.2 Je suis plus susceptible de connaître un problème de santé mentale que d'autres personnes SUS2
Très en accord 1 */ En accord* 2 */ Ni d'accord ni pas d'accord* 3 */ Pas d'accord* 4 */ Pas du tout d'accord* 5

3.3 Je pense que j'aurai un problème de santé mentale à un moment ou un autre de ma vie SUS3
Très en accord 1 */ En accord* 2 */ Ni d'accord ni pas d'accord* 3 */ Pas d'accord* 4 */ Pas du tout d'accord* 5

3.4 Mes antécédents familiaux rendent plus probable l'apparition d'un problème de santé mentale SUS4
Très en accord 1 */ En accord* 2 */ Ni d'accord ni pas d'accord* 3 */ Pas d'accord* 4 */ Pas du tout d'accord* 5

<u>Sévérité perçue</u>

3.5 Un problème de santé mentale peut affecter sérieusement ma vie de tous les jours SEV1
Très en accord 1 */ En accord* 2 */ Ni d'accord ni pas d'accord* 3 */ Pas d'accord* 4 */ Pas du tout d'accord* 5

3.6 Un problème de santé mentale peut avoir des répercussions sur ma famille, mon conjoint, mes amis SEV2
Très en accord 1 */ En accord* 2 */ Ni d'accord ni pas d'accord* 3 */ Pas d'accord* 4 */ Pas du tout d'accord* 5

3.7 Un problème de santé mentale peut bouleverser complétement ma vie SEV3
Très en accord 1 */ En accord* 2 */ Ni d'accord ni pas d'accord* 3 */ Pas d'accord* 4 */ Pas du tout d'accord* 5

3.8 Les problèmes de santé mentale ont des conséquences irréversibles SEV4
Très en accord 1 */ En accord* 2 */ Ni d'accord ni pas d'accord* 3 */ Pas d'accord* 4 */ Pas du tout d'accord* 5

<u>Avantages perçus</u>

3.9 Une thérapie peut aider à surmonter un problème de santé mentale AVA1
Très en accord 1 */ En accord* 2 */ Ni d'accord ni pas d'accord* 3 */ Pas d'accord* 4 */ Pas du tout d'accord* 5

3.10 Une thérapie peut éviter qu'un problème de santé mentale s'aggrave AVA2
Très en accord 1 */ En accord* 2 */ Ni d'accord ni pas d'accord* 3 */ Pas d'accord* 4 */ Pas du tout d'accord* 5

3.11 Une thérapie peut améliorer mes relations avec les autres AVA3
Très en accord 1 */ En accord* 2 */ Ni d'accord ni pas d'accord* 3 */ Pas d'accord* 4 */ Pas du tout d'accord* 5

3.12 Une thérapie peut changer en mieux ma vie AVA4
Très en accord 1 */ En accord* 2 */ Ni d'accord ni pas d'accord* 3 */ Pas d'accord* 4 */ Pas du tout d'accord* 5

Obstacles perçus

3.13 Faire une thérapie est une perte de temps et d'énergie OBS1
Très en accord 1 */ En accord* 2 */ Ni d'accord ni pas d'accord* 3 */ Pas d'accord* 4 */ Pas du tout d'accord* 5

3.14 J'aurais honte d'avouer que je suis une thérapie OBS2
Très en accord 1 */ En accord* 2 */ Ni d'accord ni pas d'accord* 3 */ Pas d'accord* 4 */ Pas du tout d'accord* 5

3.15 Si j'avais un problème de santé mentale, je ne saurais pas où trouver de l'aide OBS2
Très en accord 1 */ En accord* 2 */ Ni d'accord ni pas d'accord* 3 */ Pas d'accord* 4 */ Pas du tout d'accord* 5

3.16 J'aurais honte d'avouer que je suis une thérapie OBS2
Très en accord 1 */ En accord* 2 */ Ni d'accord ni pas d'accord* 3 */ Pas d'accord* 4 */ Pas du tout d'accord* 5

4 EMOTIONS (Robert Plutchik)

Indiquez le degré de vos émotions dans les deux dernières semaines :

	Très bas 1	Bas 2	Moyen 3	Elevé 4	Très élevé 5
**4.1 Joie** JOI					
**4.2 Peur** PEUR					
**4.3 Dégoût** DEG					
**4.4 Tristesse** TRIST					

**4.5 Colère** COL					
**4.6 Surprise** SUR					
**4.7 Confiance** CONF					
**4.8 Anticipation** ANT					

5 COPING STRATEGIES (the Ways of Coping strategy Questionnaire)

Indiquez quelle est la fréquence des actions suivantes lors d'un moment de difficulté émotionnelle

	Pas du tout fréquent 1	Pas Fréquent 2	Moyen 3	Fréquent 4	Très fréquent 5
**5.1 J'essaie de penser à quelque chose d'agréable** AGR					
**5.2 Je repense à des moments agréables du passé** PAS					
**5.3 Je pense à des personnes avec lesquelles j'aime être** PERS					
**5.4 Je pense aux choses que j'aime faire** FAIR					
**5.5 Je fais quelque chose qui me plait comme lire un livre, regarder un film…** PLAIT					
**5.6 Je ne pense pas à la douleur** DOUL					

5.7 **Je fais comme si la douleur n'était pas là** LA					
5.8 **J'essaie de prendre de la distance par rapport à la douleur** DIS					
5.9 **J'essaye de ne pas y penser comme si c'était mon état d'âme** PENS					
5.10 **J'imagine que la douleur est en dehors de mon corps** DEHORS					
5.11 **Je me dis d'être courageux et de continuer malgré la douleur** COUR					
5.12 **Je me dis que je peux dominer ma douleur** DOM					
5.13 **Je me dis que je ne peux pas laisser la douleur gêner ce que j'ai à faire** GENE					
5.14 **Même si je me sens mal, je continue à faire ce que j'ai à faire** CONTIN					
5.15 **Je prends des médicaments** MEDOC					

5.16 Je me fais aider AIDE					
5.17 J'essaye de penser à l'avenir AVEN					
5.18 Je fais confiance aux professionnels de santé CONFI					
5.19 Je fais confiance à mon entourage (famille, amis…) ENTOUR					

6 RECHERCHE D'AIDE

Si vous aviez un problème de santé mentale, chercheriez-vous un soutien auprès des personnes suivantes ?

	**Oui** 1	**Peut-être** 0	**Non** 2
6.1 **Mère, Père (ou tuteur légal)** SOUT_PAREN			
6.2 **Frère, sœur, demi-frère, demi-sœur** SOUT_FRE_SOE			
6.3 **Autre membre de la famille** SOUT_AUT_FAM			
6.4 **Ami proche** SOUT_AMI			
6.5 **Enseignant, tuteur (ou un autre adulte qui travaille dans mon université)** SOUT_ENS			
6.6 **Prêtre, rabbin, imam (ou un autre chef spirituel ou religieux)** SOUT_RELIG			

**6.7 Médecin généraliste** SOUT_DOC			
**6.8 Professionnel de santé mentale (p. ex psychologue, psychiatre)** SOUT_PSY			

7 EVALUATION DU JEU

7.1 Le jeu, est-il amusant ? JEU-1 Echelle de 0 (pas amusant du tout) à 10 (très amusant)

7.2 Le contenu du jeu, est-il intéressant ? JEU-2 Echelle de 0 (pas intéressant du tout) à 10 (très intéressant)

7.3 Le contenu du jeu, est-il adapté à la cible ? JEU-3 Echelle de 0 (pas adapté du tout) à 10 (très adapté)

7.4 Quel est le degré de facilité d'utilisation du jeu (instructions claires, compréhension des consignes, etc.) ? JEU-4 Echelle de 0 (pas facile du tout) à 10 (très facile)

7.5 Comment jugez-vous le graphisme du jeu ? JEU-5 Echelle de 0 (pas attractif du tout) à 10 (très attractif)

7.6 L'objectif du jeu, est-il clair ? JEU-6 Echelle de 0 (pas clair du tout) à 10 (très clair)

7.7 Conseillerez-vous à votre entourage de participer à ce jeu ? JEU-7
- Absolument pas 1
- Non 2
- Peut-être 3
- Oui 4
- Absolument oui 5

7.8 Payeriez-vous pour jouer à cette Escape Room ? JEU-8
- Oui 1
- Peut-être 2
- Non 3

7.9 Ce jeu, est-il susceptible d'accroître la prise de conscience de l'importance d'aborder la thématique de la santé mentale ? JEU-9
- Absolument pas 1
- Non 2
- Peut-être 3

- Oui 4
- Absolument oui 5

7.10 Ce jeu, est-il susceptible d'augmenter les connaissances sur la santé mentale ? JEU-10
- Absolument pas 1
- Non 2
- Peut-être 3
- Oui 4
- Absolument oui 5

7.11 Ce jeu, est-il susceptible de rendre plus positives les attitudes à l'égard de la santé mentale ? JEU-11
- Absolument pas 1
- Non 2
- Peut-être 3
- Oui 4
- Absolument oui 5

7.12 Ce jeu, est-il susceptible d'accroître les intentions/motivations à chercher de l'aide par rapport à sa propre santé mentale ? JEU-12
- Absolument pas 1
- Non 2
- Peut-être 3
- Oui 4
- Absolument oui 5

7.13 Ce jeu, est-il susceptible d'améliorer la santé mentale de ses utilisateurs ? JEU-13
- Absolument pas 1
- Non 2
- Peut-être 3
- Oui 4
- Absolument oui 5

7.14 Quel est votre note finale du jeu ? JEU-14

1 ★
2 ★★
3 ★★★
4 ★★★★
5 ★★★★★

SUIVI A SIX MOIX

7.15 Ai-je pu mettre en pratique ce que j'ai appris pendant le jeu ? PRAT

-Oui 1
-Partiellement 3
-Non 2

7.16 Ai-je pu mettre en pratique ce que j'ai appris pendant le jeu ? PRAT

-Oui 1

-Partiellement 3

-Non 2

7.17 Le jeu m'a-t-il appris des stratégies pour faire face à des périodes de détresse ?

- Oui 1
- Partiellement 3
- Non 2
