# Supplementary material for: Escape Game to Promote Students’ Mental Health Outcomes in the Aftermaths of COVID-19 Pandemic: Protocol for a Mixed Methods Study Evaluating a Cocreated Intervention
Source: JMIR Res Protoc. 2025 Apr 2;14:e64068. doi: 10.2196/64068 (PMC12004027; doi:10.2196/64068)
Supplement: Multimedia Appendix 2 [file resprot_v14i1e64068_app2.pdf]

Appendix 2

a) Interview Guide – “Mail” Group

Before starting the interview:

o Introduce yourself
o Restate the objectives of the study
o Specify the possibility of obtaining the recording/transcriptions/feedback on the results
o Specify the possibility of using informal language given the initial approach of the project focused on an informal and friendly dynamic
o Make sure you are calm
o Ask for permission to start recording
o Specify that the recording is starting

Hello,
My name is XXX and I am part of the EscapeCovid project team. Thank you for being here today and for agreeing to participate in the study.
You have received an informational email to help you promote and take care of your mental health. Today, we would like to collect your opinion and perception of this information as well as the impact it may have had on your knowledge of mental health.
This interview will last about 30 minutes. It is important to emphasize that it is completely anonymous and that there are no right or wrong answers.
Before we begin, would you agree that our exchange be recorded? This will only be used for practical purposes and to faithfully transcribe your words, and the information will only be used for this research. In this case, shall I start recording?

Topics	General instructions	Follow-ups
1.General presentation of the person interviewed	Can you tell me about yourself?	Age<br>Educational background: level and field of study<br>Motivations for participating in the study<br>Opinions on Escape Games (virtual/real; in general; in health; feedback from previous experience)
2.Mental well-being and impact of the email	How are you doing right now?<br>What impact could this email have had on your knowledge related to mental health?	Understanding mental health problems (depression and its symptoms, description of two concepts: stress/anxiety)<br>Understanding emotions and their management: coping<br>Changing beliefs about mental health

		Seeking help (sources of information, mental health care: consulting a mental health professional)
3. Perspectives and impressions on the email	Would you like to keep this email as a support to use later?	Satisfaction/dissatisfaction (content: readability, level of difficulty/form)<br>Suggestion for improvement (content, form)

Before closing the interview:

Is there anything you would like to add?
Thank you again for your contribution to our study and for taking the time to share this information with me. Now that your participation in the study is officially complete, we will get back to you to send you your gift cards to thank you.

b) Interview Guide – "Game" Group

Before starting the interview:

o Introduce yourself
o Restate the objectives of the study
o Specify the possibility of obtaining the recording/transcriptions/feedback on the results
o Specify the possibility of using informal language given the initial approach of the project focused on an informal and friendly dynamic
o Make sure you are calm
o Ask for permission to start recording
o Specify that the recording is starting

Hello,
My name is XXX and I am part of the EscapeCovid project team. Thank you for being here today and for agreeing to participate in the study.
You participated in an online *Escape Game* session that allowed us to have initial elements to evaluate our game. Today, we would like to gather your perception, your experience with this game as well as the impact it may have had on your knowledge of mental health.
This interview could will last about 30 minutes. It is important to emphasize that it is completely anonymous and that there are no right or wrong answers.
Before we begin, would you agree that our exchange be recorded? This will only be used for practical purposes in order to faithfully transcribe your words, and the information will only be used as part of this research. In this case, shall I start the recording?

**Topics**	**General instructions**	**Follow-ups**
1.General presentation of the person interviewed	Can you tell me about yourself?	Age<br>Educational background: level and field of study<br>Motivations for participating in the study<br>Opinions on *Escape Games* (virtual/real; in general; in health; feedback from previous experience)
2.Mental well-being and impact of the game	How are you doing right now?<br>What impact could this game have had on your knowledge related to mental health?	Understanding mental health problems (depression and its symptoms, description of two concepts: stress/anxiety)<br>Understanding emotions and their management: coping<br>Changing beliefs about mental health<br>Seeking help (sources of information, mental health care: consulting a mental health professional)

3. Perspectives and impressions on the game	Would you like to keep this game as a support to use later?	

Before closing the interview:

Is there anything you would like to add?
Thank you again for your contribution to our study and for taking the time to share this information with me. Now that your participation in the study is officially complete, we will get back to you to send you your gift cards to thank you.
